# Mendelian randomization suggests a causal relationship between gut microbiota and nonalcoholic fatty liver disease in humans

**DOI:** 10.1097/MD.0000000000037478

**Published:** 2024-03-22

**Authors:** Xiangyi Dai, Kaiping Jiang, Xiaojun Ma, Hongtao Hu, Xiaoai Mo, Kaizhou Huang, Qunfang Jiang, Ying Chen, Chonglin Liu

**Affiliations:** a Eighth Clinical College, Guangzhou University of Chinese Medicine, Foshan, China; b Department of Hepatology, Foshan Hospital of Traditional Chinese Medicine, Foshan, China.

**Keywords:** genetics, gut microbiota, Mendelian randomization, nonalcoholic fatty liver disease, SNPs

## Abstract

Targeting the gut microbiota is an emerging strategy to treat nonalcoholic fatty liver disease (NAFLD). Nonetheless, the causal relationship between specific gut microbiota and NAFLD remains unclear. We first obtained genome-wide association study statistics on gut microbiota and NAFLD from publicly available databases. We then performed the Mendelian randomization (MR) analysis to determine the potential causal relationship between the gut microbiota and NAFLD by 5 different methods, and conducted a series of sensitivity analyses to validate the robustness of the MR analysis results. Furthermore, we investigated the direction of causality by bidirectional MR analysis. For 211 gut microbiota, 2 MR methods confirmed that phylum Tenericutes, class Deltaproteobacteria and class Mollicutes were significantly associated with the risk of NAFLD. Heterogeneity (*P* > .05) and pleiotropy (*P* > .05) analyses validated the robustness of the MR results. There was no causal effect of NAFLD on these bacterial taxa in the reverse MR analysis. We identified specific gut microbiota with causal effects on NAFLD through gene prediction, which may provide useful guidance for targeting the gut microbiota to intervene and treat NAFLD.

## 1. Introduction

nonalcoholic fatty liver disease (NAFLD) is the most widespread hepatic disease, with a reported global prevalence of approximately 25%.^[1]^ Without intervention, NAFLD can progress to nonalcoholic steatohepatitis and even cirrhosis and hepatocellular carcinoma.^[2]^ Because of the lack of obvious early symptoms, 20% of patients have worsened to advanced fibrosis or even hepatocellular carcinoma prior to the first diagnosis.^[3]^ Early diagnosis and timely intervention are thus important for delaying or controlling NAFLD progression. Unfortunately, there are few FDA-approved drugs available for the direct treatment of NAFLD because the pathophysiological mechanism of NAFLD is not completely understood.^[4]^ Currently, the treatment of NAFLD is mainly based on lifestyle improvement, supplemented by anti-inflammatory and antioxidant drugs, but the results are not satisfactory due to poor patient compliance.^[5]^ Therefore, controlling the development of NAFLD by modulating the gut microbiota is an emerging treatment strategy.^[6]^

The gut microbiota, being the “second genome” of the human body, is closely related to metabolic disorders, immune dysregulation and even cancer development.^[7]^ The hepatic portal vein connects the liver directly to the intestine, so gut microbiota and their metabolites that enter the liver to cause or worsen an array of liver diseases.^[8]^ Therefore, the gut microbiota holds great prospects as a potential therapeutic target for NAFLD.^[9]^ Modulating the gut microbiota, such as probiotic therapy, fecal microbiota transplantation and microbiome-targeted therapies, is an emerging research trend to intervene and treat NAFLD.^[10]^ Previous animal studies have demonstrated the gut dysbiosis in high-fat diet (HFD) induced NAFLD mice disrupts the intestinal barrier, and thereby permits the transfer of the gut microbiome and its metabolites to the liver, causing endotoxemia that results in progression of NAFLD.^[11]^ Although the causality between gut microbiota and NAFLD has been demonstrated in animals, the role in humans remains less clear and warrants further investigation.^[12]^ In addition to this, most of the previous research exploring gut microbiota associated with NAFLD in humans were clinical observational studies that were influenced by many confounders. Thus, the gut microbiota profiles associated with NAFLD reported in different studies often vary widely or even contradict each other due to the effects of the populations studied, small sample sizes, dietary patterns, medication history and detection methods.^[13]^

In this context, Mendelian randomization (MR) is an emerging approach for investigating the causal role of the gut microbiota in the development of diseases through genetic prediction, such as metabolic diseases,^[14]^ autoimmune diseases,^[15]^ chronic kidney diseases,^[16]^ and psychiatric disorders.^[17]^ In the MR study, single nucleotide polymorphisms (SNPs) that are strongly associated with exposure are selected as an efficient alternative for exposure to explore the causal role between exposure and outcome, thereby minimizing the effect of confounders and reverse causality.^[18,19]^ In this study, we aimed to explore the specific gut microbiota causally associated with NAFLD from the genetic perspective, which may provide candidate bacterial taxa for future treatment of NAFLD by targeting the gut microbiota.

## 2. Methods

### 2.1. Study design

**Figure 1.**
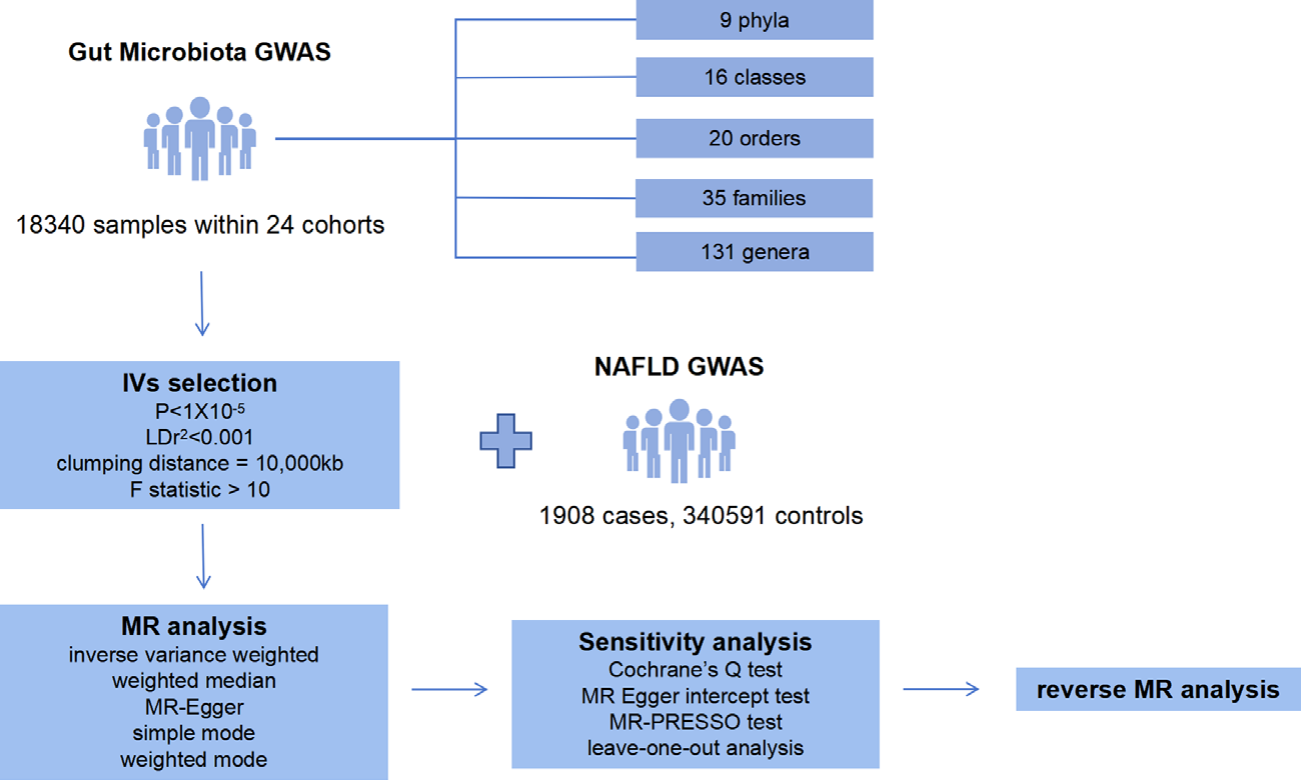
The study design of the present Mendelian randomization study of the associations of gut microbiota and nonalcoholic fatty liver disease. GWAS = genome-wide association study, IVs = instrument variables, LD = linkage disequilibrium, MR = Mendelian randomization, MR-PRESSO = Mendelian Randomization Pleiotropy RESidual Sum and Outlier, NAFLD = nonalcoholic fatty liver disease.

The main steps in the conduct of this study are shown in Figure 1. This study is based on 3 assumptions: There is a significant relevance between instrument variables (IVs) and exposure factors, there is no relevance between IVs and any confounding factors (correlated lifestyle or socioeconomic factors), IVs can only affect outcome through exposure, not through other routes.^[18]^

### 2.2. Data sources

The gut microbiota genome-wide association study (GWAS) statistics were used from a large human gut microbiota composition study conducted by the MiBioGen consortium (https://mibiogen.gcc.rug.nl), which included 24 cohorts (18,340 participants).^[20]^ The majority of these individuals were of European origin (N = 13,266). The GWAS study yielded 122,110 loci of variation, and recorded 211 gut microbiota. All cohorts in the study adjusted for sex and age during analysis. The GWAS statistics for NAFLD were derived from data published by FinnGen Research (https://r8.finngen.fi/) in December 2022. The diagnostic criteria for NAFLD are according to the ICD-10 standards. A total of 20,175,454 variables were obtained from 342,499 subjects through SAIGE. The final 1908 NAFLD cases and 340,591 controls of Finnish ancestry were included in the NAFLD study, after correction for sex, age, genotyping batch and first 10 principal components. The GWAS statistics were derived from databases of people of European descent, and the possibility of sample overlap was low.

### 2.3. IVs selection

To guarantee the reliability of the causal relationship, the following screening steps were undertaken to choose the eligible IVs. Consistent with previous research,^[21,22]^ we first identified SNPs that were significantly related to gut microbiota (*P* < 1 × 10^－5^); As a strong linkage disequilibrium can result in biased effects, the linkage disequilibrium between the SNPs was ruled out (R^2^ < 0.001, clumping distance = 10,000 kb); To avoid the effect of alleles on the causality result, the palindromic SNPs were excluded; We used the PhenoScanner V2^[23]^ to test whether the SNPs correlated with other phenotypes to further rule out potential pleiotropic effects; and we computed the F-statistic to determine whether there were weak IVs. If the F-statistic was < 10, the bias was considered to be caused by weak IVs.

### 2.4. Statistical analysis

All statistical analyses were conducted with the R software (version 4.2.2). The causal relation between the gut microbiota and NAFLD was analyzed with R-Pack “TwoSample MR” and “MRPRESSO.”

#### 2.4.1. MR analysis.

To detect the causal effect between each gut microbiota and NAFLD, we conducted MR analysis in 5 different ways, including inverse variance weighted (IVW), weighted median (WM), MR-Egger, simple mode and weighted mode. The IVW assumes that all instrumental variables meet the assumptions of MR, providing a reliable result of causality, and is therefore often used as the primary method of analysis.^[24]^ The WM can give an unbiased assessment if up to 50% of IVs are valid. Since, not all included genetic variants are valid IVs, the WM method has better finite-sample Type 1 error rates than the IVW method.^[25]^ The MR Egger can provide unbiased assessment and did not dependent on pleiotropy influences.^[26]^ This method can be considered in combination in sensitivity analyses.^[27]^ The simple mode is an unweighted model-based estimator that provides robustness for pleiotropy.^[28,29]^ The weighted model is effective when most of the IVs are valid, even if the other IVs in the method do not satisfy the MR assumptions.^[28,30]^ Results were considered reliable if a significant causal relationship between gut microbiota and NAFLD was established by 2 or more MR methods.

#### 2.4.2. Sensitivity analysis and reverse MR analysis.

To determine the robustness of the results, a range of sensitivity analyses were carried out. Cochrane *Q* test (IVW) and Rucker *Q* test (MR-Egger) were performed to assess for heterogeneity, and *P* > .05 means that there is no heterogeneity. MR Egger intercept test was conducted to detect the effect of horizontal pleiotropy, and horizontal pleiotropy was considered absent if *P* > .05. MR pleiotropy residual sum and outlier (MR-PRESSO) were used to further analyze the horizontal pleiotropy and to exclude potential outliers. Furthermore, leave-one-out method was used to pinpoint whether there was any bias due to individual IVs independently influencing the results. If there were IVs that contributed to the bias, we repeated the Mr analysis after excluding these IVs. Finally, reverse MR analysis was performed to investigate whether there was an inverse causal relationship between NAFLD and identified significant gut microbiota.

## 3. Results

### 3.1. IVs selection

The 15 unknown bacterial taxa were excluded, leaving 196 bacterial taxa, which included 5 biological classifications: 9 phyla, 16 classes, 20 orders, 32 families and 119 genera. After strict screening, the IVs for each bacterial taxa were between 5 and 12 (Table 1). All F-statistics were above 10, suggesting the absence of a weak IV (Supplementary Table 1, http://links.lww.com/MD/L911).

**Table 1:** Results of 5 Mendelian randomization methods.

Gut microbiota	Method	nSNPs	OR	95%CI	*P* value
Phylum Tenericutes	WM	12	1.60	1.077–2.389	.020
	IVW	12	1.37	1.028–1.838	.032
Class Deltaproteobacteria	WM	12	1.61	1.020–2.539	.041
	IVW	12	1.65	1.162–2.349	.005
Class Mollicutes	WM	12	1.60	1.088–2.366	.017
	IVW	12	1.37	1.028–1.838	.032
Order *Desulfovibrionales*	IVW	11	1.62	1.127–2.339	.009
Order *Enterobacteriales*	IVW	7	0.57	0.369–0.889	.013
Family Desulfovibrionaceae	IVW	9	1.73	1.164–2.571	.007
Family Enterobacteriaceae	IVW	7	0.57	0.369–0.889	.013
Family Streptococcaceae	WM	11	0.54	0.335–0.883	.014
Genus Hungatella	IVW	5	1.37	1.030–1.813	.030
Genus Senegalimassilia	IVW	5	0.66	0.445–0.969	.034
Genus Streptococcus	WM	12	0.56	0.346–0.903	.018

CI = confidence interval, IVW = inverse variance weighted, OR = odds ratio, SNP = single nucleotide polymorphism, WM = weighted median.

### 3.2. MR analysis

As shown in Table 1, we analyzed the causal relationships between 196 bacterial taxa and NAFLD using 5 MR methods, and the results indicate that 11 causal relationships between bacterial taxa and NAFLD were identified using at least 1 MR method. Among these, 3 bacterial taxa associated with NAFLD were cross-validated by the IVW and WM results. Specifically, phylum Tenericutes (IVW OR = 1.37 (1.028–1.838), *P* = .032; WM OR = 1.60 (1.077–2.389), *P* = .020), class Deltaproteobacteria (IVW OR = 1.65 (1.162–2.349), *P* = .005; WM OR = 1.61 (1.020–2.539), *P* = .041) and class Mollicutes (IVW OR = 1.37 (1.028–1.838), *P* = .032; WM OR = 1.60 (1.088–2.366), *P* = .017) were strongly associated with the risk of NAFLD. Our discussion on causality also focuses on these 3 taxa. In addition, causal associations of orders (Desulfovibrionales, Enterobacteriales), families (Desulfovibrionaceae, Enterobacteriaceae, Streptococcaceae), genera (Hungatella, Senegalimassilia, Streptococcus) on NAFLD were also suggested (Supplementary Fig. 1, http://links.lww.com/MD/L910).

### 3.3. Sensitivity analysis and reverse MR analysis

**Figure 2.**
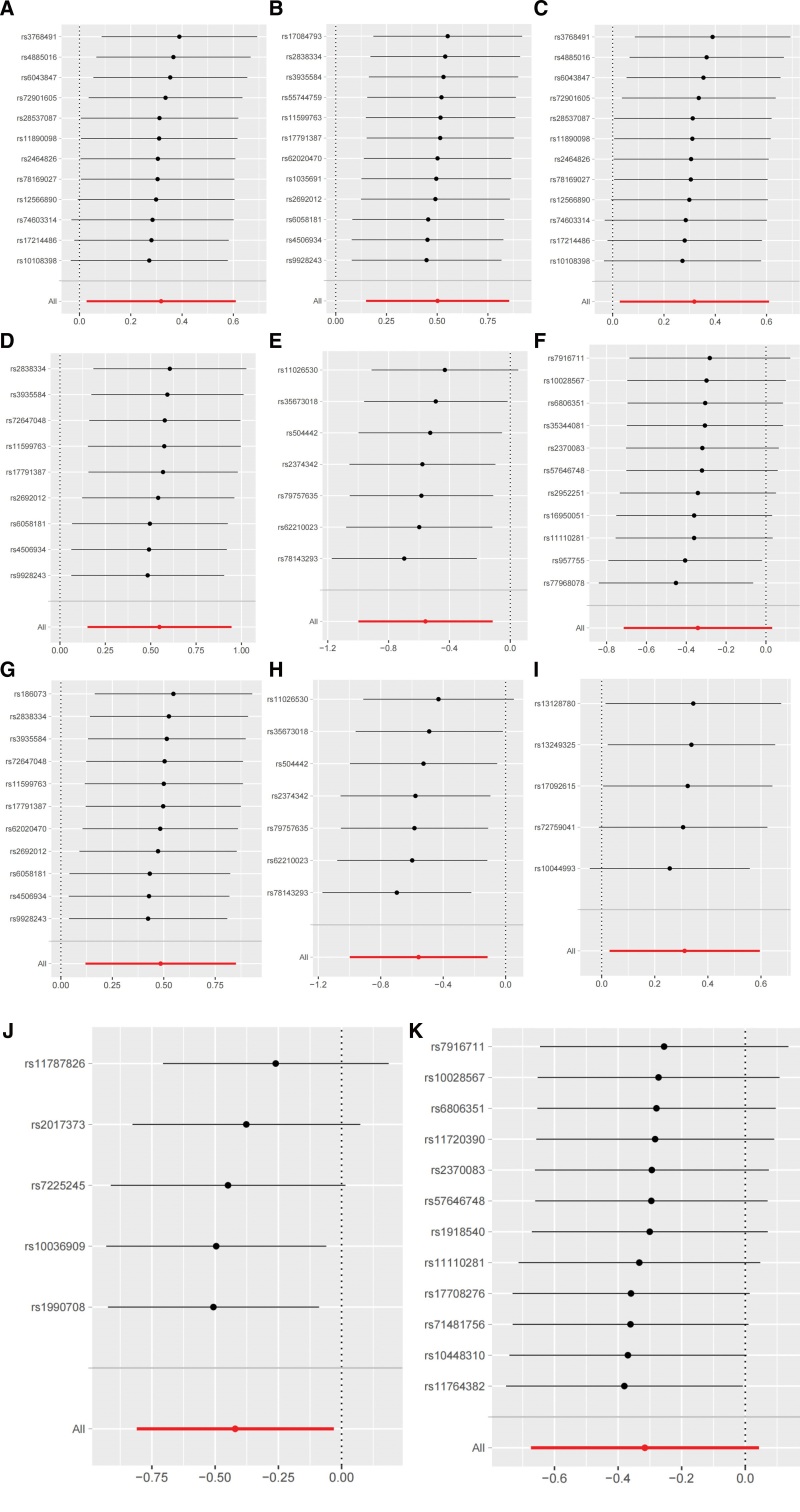
Leave-one-out plots for the causal association between gut microbiota and nonalcoholic fatty liver disease. (A) Phylum Tenericutes; (B) Class Deltaproteobacteria; (C) Class Mollicutes; (D) Family Desulfovibrionaceae; (E) Family Enterobacteriaceae; (F) Family Streptococcaceae; (G) Order *Desulfovibrionales*; (H) Order *Enterobacteriales*; (I) Genus Hungatella; (J) Genus Senegalimassilia; (K) Genus Streptococcus.

As shown in Table 2, we performed Cochrane *Q* test and Rucker *Q* test to assess heterogeneity, and all *P* values were > .05, suggesting that the probability of heterogeneity was low for the selected SNPs. All *P* values for the MR-Egger intercept test and the MR-PRESSO global test were also > 0.05, which indicates that our results are not expected to be biased by horizontal pleiotropy. And, the MR-PRESSO outlier test did not identify any outliers, and the results further confirmed the robustness of the causality. We also conducted the leave-one-out analysis for the detected potential effect IVs, and the results indicate that no IV independently affects the robustness of the causal relationship (Fig. 2). Finally, the results of the reverse MR analysis indicated no causal effect of NAFLD on the identified gut microbiota.

**Table 2:** Results of heterogeneity and horizontal pleiotropy.

Gut microbiota	Heterogeneity test		MR-PRESSO		MR-Egger
	Q-P (MR Egger)	Q-P (IVW)	MR-PRESSO global test	Outlier-corrected	Egger intercept test
Phylum Tenericutes	0.714	0.780	0.806	NA	0.745
Class Deltaproteobacteria	0.968	0.976	0.987	NA	0.607
Class Mollicutes	0.714	0.780	0.782	NA	0.745
Family Desulfovibrionaceae	0.934	0.945	0.984	NA	0.542
Family Enterobacteriaceae	0.555	0.642	0.34	NA	0.608
Family Streptococcaceae	0.619	0.693	0.557	NA	0.690
Order *Desulfovibrionales*	0.965	0.949	0.962	NA	0.352
Order *Enterobacteriales*	0.555	0.642	0.327	NA	0.608
genus Hungatella	0.760	0.876	0.902	NA	0.849
Genus Senegalimassilia	0.570	0.499	0.539	NA	0.329
Genus Streptococcus	0.849	0.864	0.733	NA	0.477

IVW = inverse variance weighted, MR-PRESSO = MR pleiotropy residual sum and outlier.

## 4. Discussion

This study comprehensively explore the causal role of the gut microbiota on NAFLD in humans from a genetic perspective that is not affected by confounders and reverse causality. This makes the results more authentic and the causal explanation more reliable, which may be a helpful contribution to the study of the pathogenesis of NAFLD and provide some guidance for future gut microbiota-targeted approach in NAFLD. In this study, the phylum Tenericutes, the class Deltaproteobacteria and the class Mollicutes were demonstrated to have a significant causal relationship with NAFLD by 2 different MR methods, which provide candidate bacterial taxa for future treatment of NAFLD by targeting the gut microbiota.

Among the bacterial taxa with causal effects on NAFLD, class Deltaproteobacteria and its sub taxa, order *Desulfovibrionales* and family Desulfovibrionaceae, all increase the risk of NAFLD. The causal role of Deltaproteobacteria in the pathogenesis of NAFLD was cross-validated using 2 methods. The class Deltaproteobacteria is the fourth classified class of the phylum Proteobacteria and comprises a number of bacteria for dissimilatory sulphate reduction.^[31,32]^ It has been found that Deltaproteobacteria abundance is significantly increased in type 2 diabetic mice.^[33]^ The abundance of the class Deltaproteobacteria was reported to be negatively associated with propionate levels in people with metabolic syndrome.^[34]^ Previous studies have demonstrated that the gut microbiota can be involved in the regulation of NAFLD through its metabolite short chain fatty acids.^[8]^ Thus, we speculate that the class Deltaproteobacteria may perturb liver metabolism and immune function in NAFLD by affecting propionate in short chain fatty acids.^[35]^ However, the specific mechanism is still unclear and further research into the possible pathogenic effects of the class Deltaproteobacteria in NAFLD is warranted. According to our study, the results of the IVW suggest that *Desulfovibrionales* and Desulfovibrionaceae also lead to an increased risk of NAFLD. In line with our findings, numerous reports have shown the important contribution of Desulfovibrio to the pathogenesis of NAFLD. In animal studies, the mouse model of HFD-induced NAFLD has been shown to have an increased abundance of Desulfovibacteriaceae.^[36,37]^ In humans, it has also been reported that BMI and Desulfovibrio are positively associated in overweight subjects.^[38]^ Mechanistically, a study found that in HFD-induced mice, Desulfovibrio piger increases intestinal permeability and the expression of the scavenge receptor CD36 in the liver, which contributed to the development of NAFLD.^[39]^ Our results provide genetic evidence to support the causal role of these gut microbiota in NAFLD. Combined with the results of previous studies, Deltaproteobacteria and its child taxa, Desulfovibrio may be potential therapeutic targets for NAFLD.

Several studies have revealed that saturated fatty acid (SFA)-induced lipotoxicity is a central factor in the development of NAFLD.^[40]^ Reportedly, the phylum Tenericutes are important hydrogenating bacteria, strongly related to body weight.^[41]^ Moreover, the Tenericutes was found to have a role in converting polyunsaturated fatty acids to SFAs by biohydrogenation.^[42]^ Therefore, we speculate that the Tenericutes causes the accumulation of SFAs by enhancing the biohydrogenation, leading to increased the risk of NAFLD. Our study is the first to report on the role of the phylum Tenericutes in NAFLD. Therefore, the results of our study may present a new perspective on the study of gut microbiota associated with NAFLD.

Similarly, the class Mollicutes was also regarded as a risk factor for NAFLD according to the IVW and WM methods. Some studies have found that the class Mollicutes is potentially related to diet-induced obesity.^[43]^ Significantly higher concentrations of lactate, acetate and butyrate were found in the cecums of obese mice containing large amounts of Mollicutes.^[44]^ Studies have previously shown that the lactate aggregation can exacerbate the progression of NAFLD and gut microbiota-derived acetate can promote hepatic lipogenesis.^[45,46]^ However, due to a lack of related research evidence, it is not possible to explain the exact mechanism by which the class mollicutes increase the risk of NAFLD. Given that the focus of our research is correlation analysis, we are unable to demonstrate the underlying mechanism yet. Therefore, the specific mechanism remains unclear and the possible role of the class Mollicutes needs to be further investigated.

However, there are several limitations to our study. First, the GWAS statistics used in this study were mainly from European populations, with only limited gut microbiota data from other ethnic populations, so the conclusions may not apply to other ethnic people. Second, given the limited number of SNPs in the gut microbiota GWAS data, the gut microbiota was only analyzed at the genus level rather than at a more specialized level, such as the species level. Third, it would be important to detect SNPs associated with NAFLD severity and to investigate the interaction between gut microbiota and NAFLD severity. However, we are currently unable to perform this correlation analysis due to the lack of reports on the specific SNP associations with NAFLD severity.^[47]^ Therefore, we propose that future studies should concentrate on linking gut microbiota to NAFLD severity.

## 5. Conclusions

In summary, we have carried out a comprehensive analysis of the genetic causal relationship between 211 gut microbiota and NAFLD. The results showed that the phylum Tenericutes, the class Deltaproteobacteria and the class Mollicutes were strongly associated with NAFLD. This study identified gut microbiota causally associated with NAFLD by genetic prediction, which may provide some guidance for future treatment of NAFLD by targeting the gut microbiota.

## Acknowledgments

Genetic association estimates for the study were obtained from the MiBioGen consortium and FinnGen consortium. We thank all investigators for sharing these data.

## Author contributions

**Conceptualization:** Xiangyi Dai, Kaiping Jiang, Xiaojun Ma.

**Methodology:** Kaiping Jiang.

**Project administration:** Kaiping Jiang.

**Visualization:** Xiangyi Dai.

**Writing – original draft:** Xiangyi Dai.

**Writing – review & editing:** Xiangyi Dai, Xiaojun Ma, Hongtao Hu, Xiaoai Mo, Kaizhou Huang, Qunfang Jiang, Ying Chen, Chonglin Liu.

## Supplementary Material




