# Supplementary material for: Mendelian randomization suggests a causal relationship between gut microbiota and nonalcoholic fatty liver disease in humans
Source: Medicine (Baltimore). 2024 Mar 22;103(12):e37478. doi: 10.1097/MD.0000000000037478 (PMC10957007; doi:10.1097/MD.0000000000037478)
Supplement: Supplementary file 1 [file medi-103-e37478-s001.docx]

**Supplementary Table 1** Results of IVs F-statistics (ranged between 11.44 and 98.69, all F>10).

F statistics were calculated using the following equation: F=R^2^(n-k-1)/k(1-R^2^), in

which R^2^ represents the variance explained by the IVs (each gut microbiome) and n represents the sample size. R^2^ was estimated by minor allele frequency (MAF) and β value, using the equation: R^2^ =2×MAF ×(1−MAF) × β^2^ .

| Phylum Tenericutes | | | | | | |  |  |  |  |
| --- | --- | --- | --- | --- | --- | --- | --- | --- | --- | --- |
|  | SNP | beta.exposure | pval.exposure | af_alt | Rsquare | F statistics |  |  |  |  |
| 1 | rs10108398 | 0.0769142 | 1.09E-06 | 0.274828 | 0.002358007 | 33.80865039 |  |  |  |  |
| 2 | rs11890098 | 0.074438 | 9.57E-07 | 0.278069 | 0.002224681 | 31.89278153 |  |  |  |  |
| 3 | rs12566890 | -0.101147 | 3.65E-06 | 0.131239 | 0.002332917 | 33.44807244 |  |  |  |  |
| 4 | rs17214486 | 0.06099 | 6.61E-06 | 0.325167 | 0.001632488 | 23.38929329 |  |  |  |  |
| 5 | rs2464826 | 0.0944239 | 8.39E-06 | 0.112011 | 0.001773626 | 25.41502762 |  |  |  |  |
| 6 | rs28537087 | 0.0820873 | 8.07E-06 | 0.247034 | 0.002506767 | 35.94691115 |  |  |  |  |
| 7 | rs3768491 | 0.0681052 | 4.23E-06 | 0.711194 | 0.001905394 | 27.30678868 |  |  |  |  |
| 8 | rs4885016 | 0.0819606 | 7.27E-06 | 0.866178 | 0.001557309 | 22.31049872 |  |  |  |  |
| 9 | rs6043847 | -0.114937 | 4.55E-06 | 0.0586119 | 0.001457821 | 20.88311603 |  |  |  |  |
| 10 | rs72901605 | -0.0841852 | 3.26E-06 | 0.11517 | 0.001444444 | 20.69121371 |  |  |  |  |
| 11 | rs74603314 | 0.221639 | 1.56E-06 | 0.0399751 | 0.003770461 | 54.13679018 |  |  |  |  |
| 12 | rs78169027 | -0.108283 | 5.88E-06 | 0.0621137 | 0.001366118 | 19.56768062 |  |  |  |  |
| Class Deltaproteobacteria | | | | | | |  |  |  |  |
|  | SNP | beta.exposure | pval.exposure | af_alt | Rsquare | F statistics | | | | |
| 1 | rs1035691 | 0.055176 | 9.65E-06 | 0.596821 | 0.001465117 | 20.98778861 | | | | |
| 2 | rs11599763 | 0.0544219 | 3.94E-06 | 0.592359 | 0.001430343 | 20.48893485 | | | | |
| 3 | rs16851319 | -0.0705516 | 5.68E-06 | 0.17701 | 0.001450227 | 20.77417926 | | | | |
| 4 | rs17084793 | -0.0710762 | 5.69E-06 | 0.145705 | 0.001257653 | 18.01211677 | | | | |
| 5 | rs17791387 | -0.0735881 | 1.60E-06 | 0.0951249 | 0.000932241 | 13.34721276 | | | | |
| 6 | rs2692012 | -0.110335 | 3.14E-06 | 0.94535 | 0.001257881 | 18.01538406 | | | | |
| 7 | rs2838334 | 0.0561722 | 5.45E-06 | 0.343782 | 0.001423653 | 20.39296444 | | | | |
| 8 | rs3935584 | -0.0523484 | 7.50E-06 | 0.532827 | 0.001364271 | 19.54119779 | | | | |
| 9 | rs4506934 | -0.0936514 | 3.59E-06 | 0.118377 | 0.001830665 | 26.23385191 | | | | |
| 10 | rs55744759 | -0.0779693 | 7.31E-06 | 0.112636 | 0.001215224 | 17.40371161 | | | | |
| 11 | rs6058181 | 0.0825716 | 3.40E-07 | 0.163036 | 0.001860723 | 26.66539624 | | | | |
| 12 | rs62020470 | -0.0585411 | 4.85E-06 | 0.179732 | 0.001010492 | 14.46869973 | | | | |
| Class Mollicutes | | | | | | |  |  |  |  |
|  | SNP | beta.exposure | pval.exposure | af_alt | Rsquare | F statistics |  |  |  |  |
| 1 | rs10108398 | 0.0769142 | 1.09E-06 | 0.274828 | 0.002358007 | 33.80865039 |  |  |  |  |
| 2 | rs11890098 | 0.074438 | 9.57E-07 | 0.278069 | 0.002224681 | 31.89278153 |  |  |  |  |
| 3 | rs12566890 | -0.101147 | 3.65E-06 | 0.131239 | 0.002332917 | 33.44807244 |  |  |  |  |
| 4 | rs17214486 | 0.06099 | 6.61E-06 | 0.325167 | 0.001632488 | 23.38929329 |  |  |  |  |
| 5 | rs2464826 | 0.0944239 | 8.39E-06 | 0.112011 | 0.001773626 | 25.41502762 |  |  |  |  |
| 6 | rs28537087 | 0.0820873 | 8.07E-06 | 0.247034 | 0.002506767 | 35.94691115 |  |  |  |  |
| 7 | rs3768491 | 0.0681052 | 4.23E-06 | 0.711194 | 0.001905394 | 27.30678868 |  |  |  |  |
| 8 | rs4885016 | 0.0819606 | 7.27E-06 | 0.866178 | 0.001557309 | 22.31049872 |  |  |  |  |
| 9 | rs6043847 | -0.114937 | 4.55E-06 | 0.0586119 | 0.001457821 | 20.88311603 | | | |  |
| 10 | rs72901605 | -0.0841852 | 3.26E-06 | 0.11517 | 0.001444444 | 20.69121371 | | | |  |
| 11 | rs74603314 | 0.221639 | 1.56E-06 | 0.0399751 | 0.003770461 | 54.13679018 | | | |  |
| 12 | rs78169027 | -0.108283 | 5.88E-06 | 0.0621137 | 0.001366118 | 19.56768062 | | | |  |
| Order Desulfovibrionales | | | | | | |  |  |  |  |
|  | SNP | beta.exposure | pval.exposure | af_alt | Rsquare | F statistics | | |  |  |
| 1 | rs11599763 | 0.0554862 | 2.61E-06 | 0.592359 | 0.001486835 | 21.29935821 | | |  |  |
| 2 | rs17791387 | -0.0727501 | 2.25E-06 | 0.0951249 | 0.000911129 | 13.04467959 | | |  |  |
| 3 | rs186073 | 0.0529219 | 8.74E-06 | 0.394995 | 0.001338602 | 19.17302558 | | |  |  |
| 4 | rs2692012 | -0.112244 | 2.27E-06 | 0.94535 | 0.001301784 | 18.64499573 | | |  |  |
| 5 | rs2838334 | 0.0569024 | 4.17E-06 | 0.343782 | 0.001460907 | 20.92738027 | | |  |  |
| 6 | rs3935584 | -0.0524466 | 7.20E-06 | 0.532827 | 0.001369395 | 19.61468157 | | |  |  |
| 7 | rs4506934 | -0.0953383 | 2.43E-06 | 0.118377 | 0.001897208 | 27.18925297 | | |  |  |
| 8 | rs6058181 | 0.0836765 | 2.53E-07 | 0.163036 | 0.001910853 | 27.38517159 | | |  |  |
| 9 | rs62020470 | -0.0573633 | 7.51E-06 | 0.179732 | 0.000970241 | 13.89179939 | | |  |  |
| 10 | rs72647048 | -0.0772459 | 9.00E-06 | 0.112623 | 0.001192659 | 17.08015888 | | |  |  |
| 11 | rs9928243 | -0.0544713 | 3.97E-06 | 0.47263 | 0.001479116 | 21.18861308 | | |  |  |
| Order Enterobacteriales | | | | | | |  |  |  |  |
|  | SNP | beta.exposure | pval.exposure | af_alt | Rsquare | F statistics | |  |  |  |
| 1 | rs11026530 | 0.0822408 | 9.43E-06 | 0.149878 | 0.00172355 | 24.69621902 | |  |  |  |
| 2 | rs2374342 | 0.0582927 | 4.52E-06 | 0.413656 | 0.001648353 | 23.61696656 | |  |  |  |
| 3 | rs35673018 | 0.0899642 | 7.63E-06 | 0.0895095 | 0.00131921 | 18.89490861 | |  |  |  |
| 4 | rs504442 | 0.084159 | 5.17E-06 | 0.105133 | 0.001332689 | 19.08821621 | |  |  |  |
| 5 | rs62210023 | 0.0606754 | 3.13E-06 | 0.345249 | 0.001664424 | 23.84760915 | |  |  |  |
| 6 | rs78143293 | -0.0848521 | 1.20E-06 | 0.12155 | 0.001537543 | 22.02687858 | |  |  |  |
| 7 | rs79757635 | 0.0758597 | 9.32E-06 | 0.137954 | 0.001368727 | 19.60511144 | |  |  |  |
| Family Desulfovibrionaceae | | | | | | |  |  |  |  |

|  | SNP | beta.exposure | pval.exposure | af_alt | Rsquare | F statistics |
| --- | --- | --- | --- | --- | --- | --- |
| 1 | rs11599763 | 0.0555931 | 2.50E-06 | 0.592359 | 0.00149257 | 21.38163097 |
| 2 | rs17791387 | -0.0729251 | 2.10E-06 | 0.0951249 | 0.000915518 | 13.10757047 |
| 3 | rs2692012 | -0.114231 | 1.56E-06 | 0.94535 | 0.001348282 | 19.31186407 |
| 4 | rs2838334 | 0.0571419 | 3.82E-06 | 0.343782 | 0.00147323 | 21.10417653 |
| 5 | rs3935584 | -0.0525838 | 6.78E-06 | 0.532827 | 0.001376569 | 19.71758124 |
| 6 | rs4506934 | -0.0943381 | 3.16E-06 | 0.118377 | 0.00185761 | 26.62070106 |
| 7 | rs6058181 | 0.0834742 | 2.70E-07 | 0.163036 | 0.001901625 | 27.25266448 |
| 8 | rs72647048 | -0.0770089 | 9.61E-06 | 0.112623 | 0.001185351 | 16.97538738 |
| 9 | rs9928243 | -0.0541732 | 4.48E-06 | 0.47263 | 0.001462971 | 20.95699493 |

| Family Enterobacteriaceae |
| --- |

|  | SNP | beta.exposure | pval.exposure | af_alt | Rsquare | F statistics |
| --- | --- | --- | --- | --- | --- | --- |
| 1 | rs11026530 | 0.0822408 | 9.43E-06 | 0.149878 | 0.00172355 | 24.69621902 |
| 2 | rs2374342 | 0.0582927 | 4.52E-06 | 0.413656 | 0.001648353 | 23.61696656 |
| 3 | rs35673018 | 0.0899642 | 7.63E-06 | 0.0895095 | 0.00131921 | 18.89490861 |
| 4 | rs504442 | 0.084159 | 5.17E-06 | 0.105133 | 0.001332689 | 19.08821621 |
| 5 | rs62210023 | 0.0606754 | 3.13E-06 | 0.345249 | 0.001664424 | 23.84760915 |
| 6 | rs78143293 | -0.0848521 | 1.20E-06 | 0.12155 | 0.001537543 | 22.02687858 |
| 7 | rs79757635 | 0.0758597 | 9.32E-06 | 0.137954 | 0.001368727 | 19.60511144 |

| Family Streptococcaceae | | | | | | |  |
| --- | --- | --- | --- | --- | --- | --- | --- |
|  | SNP | beta.exposure | pval.exposure | af_alt | Rsquare | F statistics | |
| 1 | rs10028567 | -0.0934027 | 3.72E-06 | 0.120424 | 0.001848142 | 26.48476709 | |
| 2 | rs11110281 | -0.130554 | 1.40E-08 | 0.0486826 | 0.001578736 | 22.61795029 | |
| 3 | rs16950051 | 0.107008 | 5.34E-06 | 0.0652692 | 0.001397196 | 20.01345426 | |
| 4 | rs2952251 | 0.0639298 | 3.72E-07 | 0.772132 | 0.001438174 | 20.60127605 | |
| 5 | rs35344081 | 0.0609349 | 2.64E-06 | 0.263011 | 0.001439452 | 20.6195991 | |
| 6 | rs6563952 | -0.0801931 | 8.71E-06 | 0.907474 | 0.001079946 | 15.46424594 | |
| 7 | rs6806351 | -0.0619209 | 6.94E-06 | 0.219111 | 0.001312073 | 18.79254953 | |
| 8 | rs76717940 | 0.150606 | 3.09E-06 | 0.0569142 | 0.002434929 | 34.9142448 | |
| 9 | rs77968078 | -0.0993013 | 7.93E-06 | 0.0603743 | 0.001118786 | 16.021033 | |
| 10 | rs7916711 | 0.0959639 | 6.33E-06 | 0.139087 | 0.002205421 | 31.61607418 | |
| 11 | rs957755 | -0.0642449 | 7.42E-06 | 0.13949 | 0.000990846 | 14.18712321 | |
| Genus Hungatella | | | | | | |  |

|  | SNP | beta.exposure | pval.exposure | af_alt | Rsquare | F statistics | |
| --- | --- | --- | --- | --- | --- | --- | --- |
| 1 | rs10044993 | -0.139547 | 8.07E-06 | 0.91428 | 0.003052336 | 43.79429384 | |
| 2 | rs13128780 | -0.149725 | 1.75E-06 | 0.188303 | 0.006852828 | 98.69921766 | |
| 3 | rs13249325 | -0.100023 | 9.69E-06 | 0.468804 | 0.004982828 | 71.63129095 | |
| 4 | rs17092615 | 0.152235 | 7.38E-06 | 0.138364 | 0.005525937 | 79.48222284 | |
| 5 | rs72759041 | -0.126025 | 3.86E-06 | 0.208712 | 0.005245965 | 75.43401076 | |
| Genus Senegalimassilia | | | | | | | |
|  | SNP | beta.exposure | pval.exposure | af_alt | Rsquare | F statistics |  |
| 1 | rs10036909 | 0.185519 | 8.05E-06 | 0.0412016 | 0.002719244 | 39.0021214 |  |
| 2 | rs11787826 | 0.081327 | 2.63E-06 | 0.408571 | 0.003196463 | 45.86882178 |  |
| 3 | rs1990708 | -0.109619 | 8.91E-06 | 0.0782013 | 0.001732414 | 24.82345704 |  |
| 4 | rs2017373 | 0.0782258 | 9.50E-06 | 0.364216 | 0.002833992 | 40.6526324 |  |
| 5 | rs7225245 | -0.079173 | 4.18E-06 | 0.568984 | 0.003074522 | 44.11359494 |  |
| Genus Streptococcus | | | | | | | |
|  | SNP | beta.exposure | pval.exposure | af_alt | Rsquare | F statistics |  |
| 1 | rs10028567 | -0.0921167 | 7.30E-06 | 0.120424 | 0.0017976 | 25.75918094 | |
| 2 | rs10448310 | -0.0517935 | 3.31E-06 | 0.359895 | 0.001235969 | 17.70117746 | |
| 3 | rs11110281 | -0.137519 | 2.58E-09 | 0.0486826 | 0.001751679 | 25.09998864 | |
| 4 | rs11720390 | 0.107024 | 3.59E-06 | 0.0625661 | 0.001343606 | 19.24480464 | |
| 5 | rs11764382 | -0.0695345 | 1.29E-06 | 0.139487 | 0.001160705 | 16.6220169 | |
| 6 | rs17708276 | -0.0793955 | 3.04E-06 | 0.108505 | 0.001219524 | 17.46537409 | |
| 7 | rs1918540 | -0.059639 | 2.44E-06 | 0.814004 | 0.001077013 | 15.42220028 | |
| 8 | rs2370083 | -0.0816836 | 9.75E-06 | 0.0639897 | 0.000799264 | 11.44182217 | |
| 9 | rs6806351 | -0.0633829 | 4.94E-06 | 0.219111 | 0.001374763 | 19.69167492 | |
| 10 | rs71481756 | 0.0931048 | 6.51E-06 | 0.0666412 | 0.001078364 | 15.44157716 | |
| 11 | rs7916711 | 0.102891 | 2.72E-06 | 0.139087 | 0.002535307 | 36.35720987 | |
