# Supplementary material for: Mendelian randomization suggests a causal relationship between gut microbiota and nonalcoholic fatty liver disease in humans
Source: Medicine (Baltimore). 2024 Mar 22;103(12):e37478. doi: 10.1097/MD.0000000000037478 (PMC10957007; doi:10.1097/MD.0000000000037478)

**Supplementary Figure 1** Forest plots for the causal association between gut microbiota and non-alcoholic fatty liver disease.

(A) Phylum Tenericutes; (B) Class Deltaproteobacteria; (C) Class Mollicutes; (D) Family Desulfovibrionaceae; (E) Family Enterobacteriaceae ; (F) Family Streptococcaceae; (G) Order Desulfovibrionales; (H) Order Enterobacteriales; (I) Genus Hungatella; (J) Genus Senegalimassilia; (K) Genus Streptococcus.


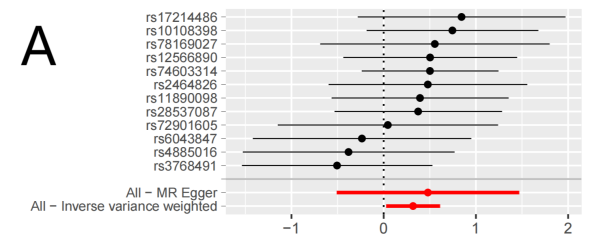

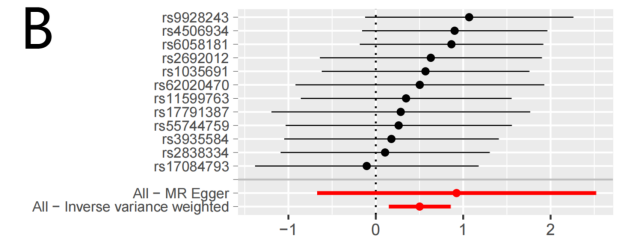


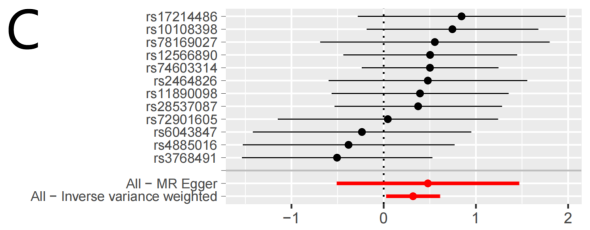

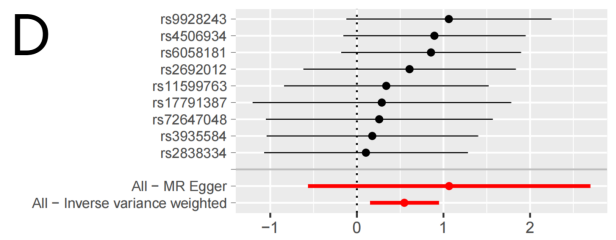


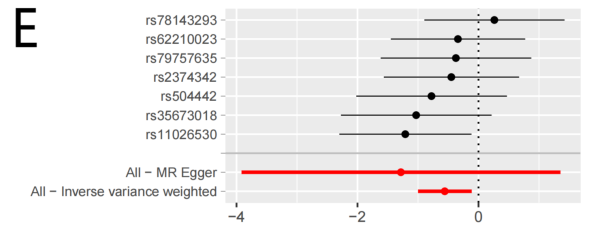

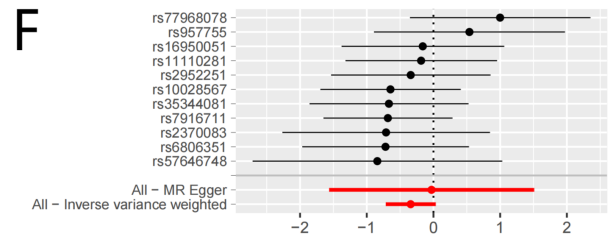


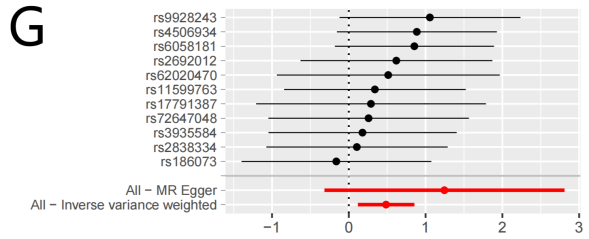

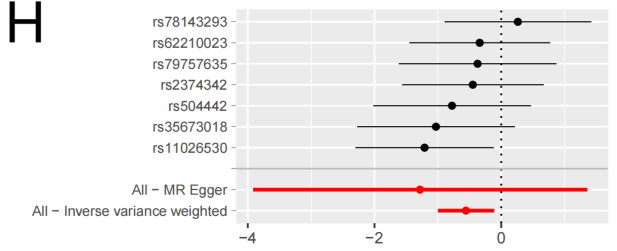


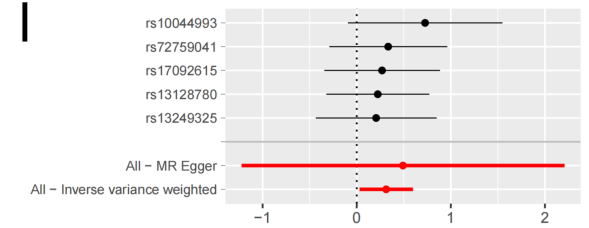

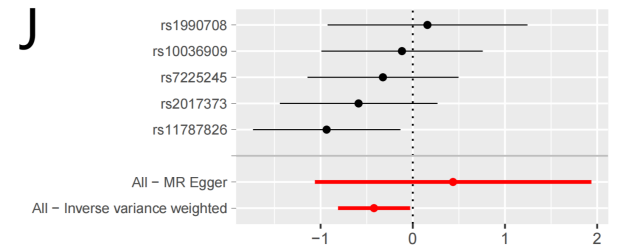


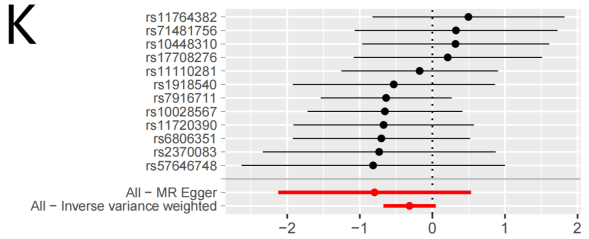

Supplement: Supplementary file 2 [file medi-103-e37478-s002.docx]
